# Artificial Intelligence and Machine Learning in Sport Research: An Introduction for Non-data Scientists

**DOI:** 10.3389/fspor.2021.682287

**Published:** 2021-12-08

**Authors:** Nader Chmait, Hans Westerbeek

**Affiliations:** Institute for Health and Sport, Victoria University, Melbourne, VIC, Australia

**Keywords:** artificial intelligence, machine learning, sports business, sports analytics, sport research, future of sports

## Abstract

In the last two decades, artificial intelligence (AI) has transformed the way in which we consume and analyse sports. The role of AI in improving decision-making and forecasting in sports, amongst many other advantages, is rapidly expanding and gaining more attention in both the academic sector and the industry. Nonetheless, for many sports audiences, professionals and policy makers, who are not particularly *au courant* or experts in AI, the connexion between artificial intelligence and sports remains fuzzy. Likewise, for many, the motivations for adopting a machine learning (ML) paradigm in sports analytics are still either faint or unclear. In this perspective paper, we present a high-level, non-technical, overview of the machine learning paradigm that motivates its potential for enhancing sports (performance and business) analytics. We provide a summary of some relevant research literature on the areas in which artificial intelligence and machine learning have been applied to the sports industry and in sport research. Finally, we present some hypothetical scenarios of how AI and ML could shape the future of sports.

## Introduction

It was in Moneyball (Lewis, 2004), the famous success storey of the Major League Baseball team “Oakland Athletics,” that using in-game play statistics came under focus as a means to assemble an exceptional team. Despite Oakland Athletics' relatively small budget, the adoption of a rigorous data-driven approach to assemble a new team led to the playoffs in the year 2002. An economic evaluation of the Moneyball hypothesis (Hakes and Sauer, 2006) describes how, at the time, a baseball hitters' salary was not truly explained by the contribution of a player's batting skills to winning games. Oakland Athletics gained a big advantage over their competitors by identifying and exploiting this information gap. It's been almost two decades since Moneyball principles, or SABRmetrics (Lewis, 2004) was introduced to baseball. SABR stands for Society for American Baseball Research and SABRmetricians are those scientists who gather the in-game data and analyse it to answer questions that will lead to improving team performance. Since the success of the Oakland Athletics, most MLB teams started employing SABRmetricians. The ongoing and exponential increase of computer processing power has further accelerated the ability to analyse “big data,” and indeed, computers increasingly are taking charge of the deeper analysis of data sets, through means of artificial intelligence (AI). Likewise, the surge in high-quality data collection and data aggregation (accomplished by organisations like Baseball Savant/StatCast, ESPN and others) are key ingredients to the spike in the accuracy and breadth of analytics that was observed in the MLB in recent years.

The adoption of AI and statistical modelling in sports has become therefore more prominent in recent years as new technologies and research applications are impacting professional sports at various levels of sophistication. The wide applicability of machine learning algorithms, combined with increasing computing processing power as well as access to more and new sources of data in recent years, has made sports organisations hungry for new applications and strategies. The overriding aim is still to make them more competitive on and off the field–in athletic and business performance. The benefits of leveraging the power of AI can, in that regard, take different forms from optimising business or technical decision making to enhancing athlete/team performance but also increasing demand for attendance at sporting events, as well as promoting alternative entertainment formats of the sport.

We next list some areas where AI and machine learning (ML) have left their footprints in the world of sports (Beal et al., 2019) and provide some examples of applications in each (some of the listed applications could overlap with one or more of the areas).

- *Game activity/analytics: match outcome modelling, player/ball Tracking, match event (e.g., shot) classification, umpire assistance, sports betting*.
- *Talent identification and acquisition: player recruitment, player performance measurement, biomechanics*.
- *Training and coaching: assessment of team formation efficacy, tactical planning, player injury modelling*.
- *Fan and business focused: measurement of a player's economic value, modelling demand for event attendance, ticket pricing optimisation (variable and dynamic), wearable and sensor design, highlight packaging, virtual and augmented reality sport applications, etc*.

The field of AI (particularly ML) offers new methodologies that have proven to be beneficial for tackling the above challenges. In this perspective paper we aim to provide sports business professionals and non-technical sports audiences, coaches, business leaders, policy makers and stakeholders with an overview of the range of AI approaches used to analyse sport performance and business centric problems. We also discuss perspectives on how AI could shape the future of sports in the next few years.

## Research on AI and ML in Sports

In this section, we will not be reviewing examples of how AI has been applied to sports for a specific application, but rather, we will look at the intersection of AI and sports at a more abstract level, discussing some research that either surveyed or summarised the application of AI and ML in sports.

One of the earliest works discussing the potential applications of artificial intelligence in sports performance, and its positive impact on improving decision-making is by Lapham and Bartlett (1995). The paper discusses how *expert systems* (i.e., a knowledge-based database used for reasoning) can be used for sports biomechanics purposes. Bartlett (2006) reviewed developments in the use of AI in sports biomechanics (e.g., throwing, shot putting, football kicking, …) to show that, at the time of writing, expert systems were marginally used in sports biomechanics despite being popular for “gait analysis” whereas Artificial Neural Networks were used for applications such as performance patterns in training and movement patterns of sports performers. An Artificial Neural Network (ANN) is a system that mimics the functionality of a human brain. ANNs are used to solve computational problems or estimate functions from a given data input, by imitating the way neurons are fired or activated in the human brain. Several (layers of) artificial neurons, known as perceptrons, are connected to perform computations which return an output as a function of the provided input (Anderson, 1995).

Bartlett (2006) predicted that multi-layer ANNs will play a big role in sports technique analysis in the future. Indeed, as we discuss later, multi-layer ANNs, now commonly referred to as Deep Learning, have become one of the most popular techniques in sports related analytics. Last but not least Bartlett (2006) described the applications of Evolutionary Computation and hybrid systems in the optimization of sports techniques and skill learning. Further discussion around the applications of AI in sports biomechanics can be found in Ratiu et al. (2010). McCabe and Trevathan (2008) discussed the use of artificial intelligence for prediction of sporting outcomes, showing how the behaviour of teams can be modelled in different sporting contests using multi-layer ANNs.

Between 2006 and 2010, machine learning algorithms, particularly ANNs were becoming more popular amongst computer scientists. This was aided by the impressive improvements in computer hardware, but also due to a shift in mindset in the AI community. Large volumes of data were made public amongst researchers and scientists (e.g., ImageNet a visual database delivered by Stanford University), and new open-source machine learning competitions were organised (such as Netflix Prize and Kaggle). It is these types of events that have shaped the adoption of AI and machine learning in many different fields of study from medicine to econometrics and sports, by facilitating access to training data and offering free open-source tools and frameworks for leveraging the power of AI. Note that, in addition to ANN, other machine learning techniques are utilised in such competitions, and sometimes these can be used in combination with one another. For instance, some of the techniques that went into the winning of the Netflix prize include singular value decomposition combined with restricted Boltzmann machines and gradient boosted decision trees.

Other examples discussing ANNs in sports include Novatchkov and Baca (2013) who discuss how ANNs can be used for understanding the quality of execution, assisting athletes and coaches, and training optimisation. However, the applications of AI to sports analytics go beyond the use of ANNs. For example, Fister et al. (2015) discussed how nature-inspired AI algorithms can be used to investigate unsolved research problems regarding safe and effective training plans. Their approach (Fister et al., 2015) relies on the notion of artificial collective intelligence (Chmait et al., 2016; Chmait, 2017) and the adaptability of algorithms to adapt to a changing environment. The authors show how such algorithms can be used to develop an artificial trainer to recommend athletes with an informed training strategy after taking into consideration various factors related to the athlete's physique and readiness. Other types of scientific methods that include Bayesian approaches have been applied to determining player abilities (Whitaker et al., 2021) but also predicting match outcomes (Yang and Swartz, 2004). Bayesian analysis and learning is an approach for building (statistical and inference) models by updating the probability for a hypothesis as more evidence or information becomes available by using Bayes' theorem (Ghosh et al., 2007).

There are numerous research papers in which AI and ML is applied to sport, and it is not our aim to comprehensively discuss these works here^1^. However, we refer to a recent survey that elaborates on this topic. Beal et al. (2019) surveyed the applications of AI in team sports. The authors summarised existing academic work, in a range of sports, tackling issues such as match outcome modelling, in-game tactical decision making, player performance in fantasy sport games, and managing professional players' sport injuries. Work by Nadikattu (2020) presents, at an abstract level, discussions on how AI can be implemented in (American) sports from enhancing player performance, to assisting coaches to come up with the right formations and tactics, to developing automated video highlights of sports matches and supporting referees using computer vision applications.

We emphasise that the application of AI in sports is not limited to topics of sports performance, athlete talent identification or the technical analysis of the game. The (off the field) business side of sports organisations is rapidly shifting towards a data driven culture led by developing profiles of their fans and their consumer preferences. As fans call for superior content and entertainment, sport organisations must react by delivering a customised experience to their patrons. This is often achieved by the use of statistical modelling as well as other machine learning solutions, for example, to understand the value of players from an economic perspective. As shown in Chmait et al. (2020a), investigating the relationship between the talent and success of athletes (to determine the existence of what is referred to as superstardom phenomenon or star power) is becoming an important angle to explore value created in sport. To provide an idea of the extent of such work, we note some sports in which the relationship between famous players/teams and their effect on audience attendance or sport consumption has been studied:

- In soccer (Brandes et al., 2008; Jewell, 2017),
- In Major League Baseball (Ormiston, 2014; Lewis and Yoon, 2016)
- In the National Basketball Association (Berri et al., 2004; Jane, 2016)
- In tennis: superstar player effect in demand for tennis tournament attendance (Chmait et al., 2020a), the presence of a stardom effect in social media (Chmait et al., 2020b), player effect on German television audience demand for live broadcast tennis matches (Konjer et al., 2017)
- And similarly, in Cricket (Paton and Cooke, 2005), Hockey (Coates and Humphreys, 2012), and in the Australian Football League (Lenten, 2012).

AI algorithms are being used in Formula 1 (F1) to improve the racing tactics of competing teams by analysing data from hundreds of sensors in the F1 car. Recent work by Piccinotti (2021) shows how artificial intelligence can provide F1 with automated ways for identifying tyre replacement strategies by modelling pit-stop timing and frequency as sequential decision-making problems.

Researchers from Tennis Australia and Victoria University devised a racket recommendation technique based on real HawkEye (computer vision system) data. An algorithm was used to recommend a selection of rackets based on movement, hitting pattern and style of the player with the aim to improve the player's performance (Krause, 2019).

Accurate and fair judging of sophisticated skills in sports like gymnastics is a difficult task. Recently, a judging system was developed by Fujitsu Ltd. The system scores a routine based on the angles of a gymnast's joints. It uses AI to analyse 3D laser sensors that capture the gymnasts' movements (Atiković et al., 2020).

Finally, it is important to note the exceptionally successful adoption of AI in board games like Chess, Checkers, Shogi and the Chinese game of GO, as well as virtual games (like Dota2 and StarCraft). In the last couple of decades, AI has delivered a staggering rise in performance in such areas to the point that machines (almost) constantly defeat human world champions. We refer to some notable solutions like Schaeffer et al. (2007) Checkers artificial algorithm, DeepBlue defeating Kasparov in Chess (Campbell et al., 2002), AlphaGo Zero defeating Lee Sedol in Go (Silver et al., 2017) (noting that AlphaZero is also unbeatable in chess) and Vinyals et al. (2019) AlphaStar in StarcraftII as well as superhuman AI for multiplayer poker (Brown and Sandholm, 2019). Commonly, in these types of games or sports, AI algorithms rely on a Reinforcement Learning approach (which we will describe later) as well as using techniques like the Monte-Carlo Search Trees to explore the game and devise robust strategies to solve and play these games. Some of the recent testbeds used to evaluate AI agents and algorithms are discussed in Hernández-Orallo et al. (2017). For a broader investigation of AI in board and virtual/computer games refer to Risi and Preuss (2020).

The rise of applying AI and ML is unstoppable and to that end, one might be wondering how AI an ML tools work and why are they different from traditional summary analytics. We touch upon these considerations in the next section.

## The Machine Learning Paradigm

To understand why ML is used in a wide range of applications, we need to take a look into the difference between recent AI approaches to learning and traditional analytics approaches. At a higher conceptual level, one can describe old or traditional approaches to sports analytics, as starting off with some set of rules that constitute the problem definition, some data that is to be processed using a program/application which will then deliver answers to the given problem. In contrast, in a machine learning/predictive analytics paradigm, the way this process works is fundamentally different. For instance, in some approaches of the ML paradigm, one typically starts by feeding the program with *answers* and corresponding *data* to a specific problem, with an algorithm narrowing down the rules of the problem. These rules are later used for making predictions and they are evaluated or validated by testing their accuracy over new (unseen) data.

To that end, machine learning is an area of AI that is concerned with algorithms that learn from data by performing some form of inductive learning. In simple terms, ML prediction could be described as a function^2^ from a set of inputs *i*_1_, *i*_2_, …, *i*_*n*_, to forecast an unknown value *y*, as follows *f*(_*w*_1_*i1_, *w*_2_*i_2_, …, *w*_n_*i_*n*_) = *y*, where *w*_*t*_ is the weight of input *t*.

Different types or approaches of ML are used for different types of problems. Some of the most popular are *supervised learning, unsupervised learning*, and *reinforcement learning*:

- In supervised learning, we begin by observing and recording both inputs (the *i*'s) and outputs (the *y*'s) of a system, for a given period of time. This data (collection of correct examples of inputs and their corresponding outputs) is then analysed to derive the rules that underly the dynamics of the observed system, i.e., the rules that map a given input to its correct output.
- Unlike the above, in unsupervised learning, the correct examples or outputs from a given system are not available. The task of the algorithm is to discover (previously unnoticed) patterns in the input data.
- In reinforcement learning, an algorithm (usually referred to as an agent) is designed to take a series of actions that maximise its cumulative payoff or rewards over time. The agent then builds a policy (a map of action selection rules) that return a probability of taking a given action under different conditions of the problem.

For a thorough introduction to the fundamentals of machine learning and the popular ML algorithms see Bonaccorso (2017). The majority of AI applications in sports are based on one or more of the above approaches to ML. In fact, in most predictive modelling applications, the nature of the output *y* that needs to be predicted or analysed could influence the architecture of the learning algorithm.

Explaining the details of how different ML techniques work is outside the scope of this paper. However, to provide an insight into how such algorithms function in layman's terms and the differences between them, we briefly present (hypothetical) supervised, unsupervised and reinforcement learning problems in the context of sports. These examples will assist the professionals but also applied researchers who work in sport to better understand the way that data scientists think so to facilitate talking to them about their approach and methodology, without requiring to dive deep into the details of the underlying analytics.

### Supervised Learning: Predicting Player Injury

Many sports injuries (e.g., muscle strain) can be effectively treated or prevented if one is able to detect them early or predict the likelihood of sustaining them. There could be many different (combinations of) reasons/actions leading to injuries like muscle strain. For example, in the Australian Football League, some of hypotheses put forward leading to muscle strain include: muscle weakness and lack of flexibility, fatigue, inadequate warm-up, and poor lumbar posture (Brockett et al., 2004). Detecting the patterns that can lead to such injuries is extremely important both for the safety of the players, and for the success and competitiveness of the team.

In a supervised learning scenario, data about the players would be collected from previous seasons including details such as the *number of overall matches and consecutive matches they played, total time played in each match, categorised by age, number of metres run, whether or not they warmed up before the match, how many times they were tackled by other players, and so on*, but more importantly, whether or not the players ended up injured and missed their next match.

The last point is very important as it is the principal difference between supervised learning and other approaches: the outcome (whether or not the player was injured) is known in the historical data that was collected from previous seasons. This historical data is then fed (with the outcome) to a machine learning algorithm with the objective of learning the patterns (combination of factors) which led to an injury (and usually assigning a probability of the likelihood of an injury given these patterns). Once these patterns are learnt, the algorithm or model is then tested on new (unseen data) to see if it performs well and indeed predicts/explains injury at a high level of accuracy (e.g., 70% of the time). If the accuracy of the model is not as required, the model is tuned (or trained with slightly different parameters) until it reaches the desired or acceptable accuracy. Note here that we did not single out a specific algorithm or technique to achieve the above. Indeed, this approach can be applied using many different ML algorithms such as Neural Networks, Decision Trees and regression models.

### Unsupervised Learning: Fan Segmentation

We will use a sport business example to introduce the unsupervised learning approach. Most sports organisations keep track of historical data about their patrons who attended their sporting events, recording characteristics such as their gender, postcode, age, nationality, education, income, marital status, etc. A natural question of interest here is to understand if the different segments of customers/patrons will purchase different categories (e.g., price, duration, class etc.) of tickets.

Some AI algorithms are designed to help split the available data, so that each data point (historical ticket sale) sits in a group/class that is similar to the other data points (other sales) in that same class given the recorded features. The algorithm will then use some sort of a *similarity* or *distance* metric to classify the patrons according to the category of tickets that they might purchase.

This is different from how supervised learning algorithms, like those discussed in the previous section, work. As we described before, in supervised learning we instruct the algorithm with the outcome in advance while training it (i.e., we classify/label each observation based on the outcome: injury or no injury, cheap or expensive seats, …). In the unsupervised learning approach, there is no such labelling or classification of existing historical data. It is the mission of the unsupervised learning algorithm to discover (previously unnoticed) patterns in the input data and group it into (two or more) classes.

Imagine the following use case where an Australian Football League club aims to identify a highly profitable customer segment within its entire set of stadium attendees, with the aim to enhance its marketing operations. Mathematical models can be used to discover (segments of) similar customers based on variations in some customer attributes within and across each segment. A popular unsupervised learning algorithm to achieve such goal is the K-means clustering algorithm which finds the class labels from the data. This is done by iteratively assigning the data points (e.g., customers) from the input into a group/class based on the characteristics of this input. The essence is that the groups or classes to which the data points are assigned to are not defined prior to exploring the input data (although the number of groups or segments can be pre-defined) but are rather dynamically formed as the K-means algorithm iterates over the data points. In the context of customer segmentation, when presenting the mathematical model (K-means algorithm) with customer data, there is no requirement to label a portion (or any of) of this data into groups in advance in order to train the model as usually done in supervised models.

### Reinforcement Learning: Simulations and Fantasy Sports

As mentioned before, in reinforcement learning, an algorithm (such as Q-learning and SARSA algorithms) learns how to complete a series of tasks (i.e., solve a problem) by interacting with an (artificial) environment that was designed to simulate the real environment/problem at hand. Unlike the case with supervised learning, the algorithm is not explicitly instructed about the right/accurate action in different states/conditions of the environment (or steps of problem it is trying to solve). But rather it incrementally learns such a protocol through reward maximisation.

In simple terms, reinforcement learning approaches represent problems using what are referred to as: an *agent* (a software algorithm), and a table of *states* and *actions*. When the agent executes an action, it transitions from one state to another and it receives a reward or a penalty (a positive or negative numerical score respectively) as a result. The reward/penalty associated with the action-state combination is then stored in the agent's table for future reference and refinement. The agent's goal is to take the action that maximises its reward. When the agent is still unaware of the expected rewards from executing a given action when at a given state, it takes a random action and updates its table following that action. After many (thousands of) iterations over the problem space, the agent's table holds (a weighted sum of) the expected values of the rewards of all future actions starting from the initial state.

Reinforcement learning has been applied to improve the selection of team formations in fantasy sports (Matthews et al., 2012). Likewise, the use of reinforcement learning is prominent in online AI bots and simulators like chess, checkers, Go, poker, StarCraft, etc.

Finally, it is important to also note the existence of genetic or evolutionary algorithms, sometimes referred to as nature/bio-inspired algorithms. While such algorithms are not typically considered to be ML algorithms (but rather search techniques and heuristics), they are very popular in solving similar types of problems tackled by ML algorithms. In short, the idea behind such algorithms is to run (parallel) search, selection and mutation techniques, by going over possible candidate solutions of a problem. The solutions are gradually optimised until reaching a local (sub-optimal) or global maximum (optimal solution). To provide a high-level understanding of evolutionary algorithms, consider the following sequence of steps:

- We start by creating (a population of) initial candidate or random strategies/solutions to the problem at hand.
- We assess these candidate solutions (using a fitness function) and assign scores to each according to how well they solve the problem at hand.
- We then pick a selection of these candidate solutions that performed best at stage two above. We then combine (*crossbreed*) these together to generate (*breed)* new solutions (e.g., take some attributes from one candidate solution and others from another candidate solution in order to come up with a new solution).
- We then apply random changes (*mutations*) to the resulting solutions from the previous step.
- We repeat the solution combination/crossbreeding process until a satisfactory solution is reached.

Evolutionary algorithms can be used as alternative means for training machine learning algorithms such as reinforcement learning algorithms and deep neural networks.

## The Future of AI in Sport

There is no doubt that AI will continue to transform sports, and the ways in which we play, watch and analyse sports will be innovative and unexpected. In fact, machine learning has drastically changed the way we think about match strategies, player performance analytics but also how we track, identify and learn about sport consumers. A Pandora's box of ethical issues is emerging and will increasingly need to be considered when machines invade the traditionally human centred and naturally talented athlete base of sport. It is unlikely that AI will completely replace coaches and human experts, but there is no doubt that leveraging the power of AI will provide coaches and players with a big advantage and lead over those who only rely on human expertise. It will also provide sport business managers with deeper, real time insights into the behaviours, needs and wants of sport consumers and in turn AI will become a main producer of sport content that is personalised and custom made for individual consumers. But human direction and intervention seems to be, at least in the near future, still essential working towards elite sport performance and strategic decision making in sport business. The sporting performance on the field is often produced as an entertainment spectacle, where the sporting context is the platform for generating the business of sport. Replacing referees with automated AI is clearly possible and increasingly adopted in various sports, because it is more accurate and efficient, but is it what the fans want?

What might the future of sport with increasingly integrated AI look like? Currently, most of the research in AI and sports is specialised. That is to provide performance or business solutions and solve specific on and off field problems. For instance, scientists have successfully devised solutions to tackle problems like player performance measurement, and quantifying the effect of a player/team on demand for gate attendance. Nevertheless, our research has not identified studies (yet) that provide a 360-degree analysis on, for example, the absolute value of an athlete by taking into account all the dimensions of his or her performance on how much business can be developed, for example in regard to ticket sales or endorsement deals.

One of the main challenges to achieve such a comprehensive analysis is mainly due to the fact that data about players and teams, and commercial data such as ticket sales and attendance numbers, are kept proprietary and are not made public to avoid providing other parties with competitive information. Moreover, privacy is an important consideration as well. Regulations about data privacy and leakage of personal identification details must be put in place to govern the use and sharing of sports (performance and consumption) data. Data ownership, protection, security, privacy and access will all drive the need for comprehensive and tight legislation and regulation that will strongly influence the speed and comprehensiveness of the adoption of AI in sport. To that end, it is worth considering privacy and confidentiality implications independently when studying the leagues' journey of AI adoption compared to that of individual teams and ultimately the individual players. Eventually, the successful adoption of AI in a sports league will likely depend on the teams in that league and their players to be willing to share proprietary data or insights with other teams in the league. Performance data of players in particular is becoming a hot topic of disputation. It may well be AI that will determine the bargaining power of players and their agents in regard to the value of their contracts. As an extension of this it will then also be AI providing the information that will determine if players are achieving the performance objectives set by coaches and as agreed to in contracts. In other words, confidentiality and ownership of league, team or player level data will become an increasing bone of legal contention and this will be reflected in the complexity of contractual agreements and possible disputes in the change rooms and on the field of play. Being in control of which data can or cannot, and will or will not, be used is at stake.

From an economic perspective, relying on artificial algorithms could increase the revenue of sports organisations and event organisers when enabled to apply efficient variable and dynamic pricing strategies and build comprehensive and deep knowledge consumer platforms. Different types of ML algorithms can be adopted to deliver more effective customer marketing via personalisation and to increase sales funnel conversion rates.

Finally, for a window on the future of data privacy, it might be useful to return to baseball where the addiction to big data started its spread across the high-performance sport industry. Hattery (2017, p. 282) explains that in baseball “using advanced data collection systems … the MLB teams compete to create the most precise injury prediction models possible in order to protect and optimise the use of their player-assets. While this technology has the potential to offer tremendous value to both team and player, it comes with a potential conflict of interest. Players' goals are not always congruent with those of the organisation: the player strives to protect his own career while the team is attempting to capitalise on the value of an asset. For this reason, the player has an interest in accessing data that analyses his potential injury risk. This highlights a greater problem in big data: what rights will individuals possess regarding their own data points?”

This privacy issue can be further extended to the sport business space Dezfouli et al. (2020) have shown how AI can be designed to manipulate human behaviour. Algorithms learned from humans' responses who were participating in controlled experiments. The algorithms identified and targeted vulnerabilities in human decision-making. The AI succeeded in steering participants towards executing particular actions. So, will AI one day be shaping the spending behaviour of sports fans by exploiting their fan infused emotional vulnerabilities and monitoring their (for example) gambling inclinations? Will AI sacrifice the health of some athletes in favour of the bigger team winning the premiership? Or is this already happening? Time will tell.

## Data Availability Statement

The original contributions presented in the study are included in the article, further inquiries can be directed to the corresponding author.

## Author Contributions

NC and HW had major contribution to the writing of this manuscript. NC contributed to the writing of the parts around artificial intelligence and machine learning and provided examples of these. HW shaped the scope of the manuscript and wrote and edited many of its sections particularly the introduction and the discussion. Both authors contributed to the article and approved the submitted version.

## Conflict of Interest

The authors declare that the research was conducted in the absence of any commercial or financial relationships that could be construed as a potential conflict of interest.

## Publisher's Note

All claims expressed in this article are solely those of the authors and do not necessarily represent those of their affiliated organizations, or those of the publisher, the editors and the reviewers. Any product that may be evaluated in this article, or claim that may be made by its manufacturer, is not guaranteed or endorsed by the publisher.

## Footnotes

^1^For conferences and published articles on AI and sports analytics see Swartz (2020).

^2^Note that such function is also found in regression techniques where the weights/coefficients are unknown. In ML, it is usually the case where both the function and its weights are unknown and are determined using various search techniques and algorithms.
